# *Jornada de Iniciação Científica* : promoting research experience among undergraduates

**DOI:** 10.31744/einstein_journal/2025EDS2

**Published:** 2025-09-29

**Authors:** Maria Clara Soares Klein, Juliana Magdalon

**Affiliations:** 1 Hospital Israelita Albert Einstein Faculdade Israelita de Ciências da Saúde Albert Einstein São Paulo SP Brazil Faculdade Israelita de Ciências da Saúde Albert Einstein, Hospital Israelita Albert Einstein, São Paulo, SP, Brazil.

Scientific initiation is a term used in Brazil to refer to Undergraduate Research Experiences (UREs). Students join a research group and develop an individual project under the guidance of a mentor, typically a faculty member, researcher or other healthcare professional with a PhD. While these experiences are intended to cultivate critical skills in data collection, analysis, and interpretation, evidence suggests that students often devote most of their time to data collection and analysis, with limited engagement in understanding the broader context of the investigation or interpreting their findings.^( 1 )^ Activities that foster deeper learning, such as discussion with mentors and peers about their research, engagement with relevant literature, as well as synthesis and presentation of their projects through poster, are highly recommended to enhance students’ research experience.^( 1 )^ In this context, participation in scientific events plays a crucial role by allowing students to formally present and discuss their research projects with peers and professionals, thereby enriching their educational and research process.

The *Jornada de Iniciação Científica* (JIC) – or Scientific Initiation Journey – is an institutional scientific symposium designed to undergraduate students at the *Faculdade Israelita de Ciências da Saúde Albert Einstein* (FICSAE). Since its inception, the JIC has been a cornerstone of the academic calendar, with its five impactful editions held in 2018, 2019, 2021, 2023 and 2025. Each edition provides students with an event to engage in research and to share their findings.

Organized by a committee composed of students and faculty, the JIC encourages undergraduates to submit abstracts of their research projects, regardless of whether results have already been obtained. This approach emphasizes the value of the scientific process itself, from its earliest stages. All submissions undergo a meticulous evaluation by at least two institutional reviewers with research experience. Only abstracts receiving a mean score of 7.0 or higher are accepted for poster presentation. Additionally, the top 10 or 12 abstracts (depending on the edition) are selected for oral presentation, offering students an opportunity to public speaking experience. During the event, all posters and oral presentations are also evaluated by at least two institutional professionals, and awards are given for the best posters and oral presentations, celebrating excellence in research. Students not currently engaged in scientific initiation are also invited to attend the JIC as non-presenting participants, promoting a culture of scientific engagement across the institution. The program also features keynote lectures on emerging topics in health research, enriching attendees’ academic and scientific knowledge.

With the expansion of FICSAE undergraduate programs, the JIC has seen a remarkable increase in the number of participants. The inaugural edition in 2018 included only students from Medicine and Nursing programs, who submitted 76 abstracts, 27 of which presented results. On the other hand, the 5th edition, held in October of 2025, received a record 236 abstracts, 96 of which presented results, from students across seven programs: Biomedical Engineering, Dentistry, Physiotherapy, Medicine, Nursing, and Nutrition. The 5th edition also featured multidisciplinary speakers, fostering dialogue on innovation and artificial intelligence, and enhancing collaboration skills essential for translating research into practice.

Since the 3rd edition in 2021, a significant milestone was introduced: the publication of accepted abstracts in a supplement of the einstein (São Paulo) journal.^( 2 , 3 )^ This initiative provides students with valuable experience in academic publishing, reinforcing their participation in all phases of the research process. In 2021, the supplement included 2 editorials and 72 abstracts, regardless of whether they reported results.^( 2 )^ In 2023, only abstracts containing results were accepted, resulting in a supplement with 55 abstracts and 2 editorials.^( 3 )^ In both editions, one of the editorials was authored exclusively by students from the organizing committee.^( 2 , 3 )^

Feedback from JIC participants has been overwhelmingly positive, emphasizing the event role in enhancing student learning, particularly in poster design and communication skills. The program fosters scientific vocation, encourages emerging talent, and promotes critical thinking. It also provides a comprehensive understanding of their research projects, while preparing themselves for presentation, as well as discussing ideas and results with peers and experts.

In summary, the JIC plays a vital role in shaping the academic and scientific journey of undergraduate students. It underscores the importance of scientific research in professional training and celebrates the development of future leaders committed to scientific advancement and societal well-being.
